# Further Exploration of Sucrose-Citric Acid Adhesive: Synthesis and Application on Plywood

**DOI:** 10.3390/polym11111875

**Published:** 2019-11-13

**Authors:** Shijing Sun, Zhongyuan Zhao, Kenji Umemura

**Affiliations:** 1 College of Material Science and Engineering, Nanjing Forestry University, Nanjing 210037, China; sunsj-611@163.com; 2 College of Furnishings and Industrial Design, Nanjing Forestry University, Nanjing 210037, China; 3 Laboratory of Sustainable Materials, Research Institute for Sustainable Humanosphere, Kyoto University, Kyoto 611-0011, Japan

**Keywords:** eco-friendly adhesive, sucrose, citric acid, plywood

## Abstract

The development of eco-friendly adhesives is a major research direction in the wood-based material industry. Previous research has already demonstrated the mixture of sucrose and citric acid could be utilized as an adhesive for the manufacture of particleboard. Herein, based on the chemical characteristics of sucrose, a synthesized sucrose-citric acid (SC) adhesive was prepared, featuring suitable viscosity and high solid content. The investigation of synthesis conditions on the bond performance showed that the optimal mass proportion between sucrose and citric acid was 25/75, the synthesis temperature was 100 °C, and the synthesis time was 2 h. The wet shear strength of the plywood bonded with SC adhesive, which was synthesized at optimal conditions and satisfied the China National Standard GB/T 9846-2015. The synthesis mechanism was studied by both ^13^C NMR analysis and HPLC, and the chemical composition manifesting caramelization reaction occurred during the synthesis process. The results of ATR FT-IR indicated the formation of a furan ring, carbonyl, and ether groups in the cured insoluble matter of the SC adhesive, which indicated dehydration condensation as the reaction mechanism between sucrose and citric acid.

## 1. Introduction

The exploitation of bio-based composites has received increasing attention [1,2], however, the most widely utilized bio-based materials are traditional wood-based materials, for instance, particleboard [3], plywood [4], and fibreboard [5]. In the wood-based material industry, resins such as urea-formaldehyde, phenol-formaldehyde, and isocyanates are widely utilized due to their excellent adhesion properties and economically satisfactory performance [6]. However, these synthetic resins usually contain volatile organic compounds (VOC), which are harmful to human health [7,8]. Most of the raw materials of the synthesis resins are derived from fossil resources, and it is predictable that the utilization of these raw materials will be inevitably restricted in the future due to the depletion of fossil resources [9]. Therefore, the development of an eco-friendly wood adhesive composed of renewable materials has become a key topic of wood science [10,11].

Previous research has already demonstrated that citric acid could be utilized as a green adhesive of wood-based materials [12,13,14]. In these studies, a citric acid water solution was sprayed on wood particles. After or without prior drying treatment, the sprayed particles were hot-pressed at 180–200 °C to fabricate particleboards. The mechanical properties and water resistance of the resulting particleboards satisfied the JIS A 5908 standard, and the reaction mechanism was considered to be the formation of carbonyl groups between citric acid and wood composition [14]. Furthermore, sucrose was added to the citric acid solution to promote bond performance [15,16]. However, although the previous citric acid-sucrose adhesive could be used for the manufacture of particleboard, it is rarely applied to the plywood production due to its low viscosity and low solid content. To overcome this limitation of the application of the citric acid-sucrose adhesion system, this study explores a synthesis method to gain a novel citric acid-sucrose adhesive with applicable viscosity, high solid content and good bond performance which satisfy the utilize requirements of plywood. 

Sucrose (β-d-fructofuranosyl α-d-glucopyranoside) is a natural disaccharide, which is produced from sugar beet or sugarcane, and its chemistry has attracted considerable interest [17]. It is used as an organic raw material in the food, beverage, and seasoning industry since it is cheap, pure, stable, and chemically reactive [18,19,20]. Based on the research of sucrose chemistry, it is found that the heating treatment can transform sucrose into an amorphous substance along with increased solution viscosity, and acid compounds usually act as catalysts in this reaction [17,20,21,22,23]. Therefore, considering the chemical properties of sucrose and the reactivity of citric acid, there is a possibility to synthesize a suitable eco-friendly adhesive for plywood. This study investigated the effects of synthesis conditions on the bond performance of sucrose-citric acid (SC) adhesive and clarified its synthesis and curing mechanisms.

## 2. Materials and Methods 

### 2.1. Materials 

Sucrose (analytical reagent) and citric acid (analytical reagent) were purchased from Sinopharm chemical reagent Co., Ltd. (Shanghai, China), and used as received without further purification. The reagents were vacuum-dried at 60 °C for 15 h until reaching a constant mass prior to usage in experiments. Poplar veneers were obtained from Zuogezhuang, Hebei Province, China.

### 2.2. Preparation of Sucrose-Citric Acid (SC) Adhesives

Sucrose and citric acid were mixed in different proportions and were poured into a three-mouthed flask with distilled water to synthesize SC adhesives with 80 wt % solid content. Three groups of adhesives were synthesized to investigate the effects of proportion, synthesis temperature, and synthesis time on the bond performance of plywood. All synthesis processes were conducted in an oil bath under 180 rpm/min mechanical stirring, and the detail information of the synthesis condition of each group are shown in Table 1. The pH values of the adhesives were measured at 30 °C using a Leici pH meter PHBJ-206 (Leici, Shanghai, China). The viscosities of the adhesives were measured by HAAKE rotational rheometer MA S60 (HAAKE CO., Karlsruhe, Germany), in this test, 2 mL of each adhesive (without prior freeze-drying) was dripped on a flat plate, the measuring geometry was C60 2°/Ti-02170027, the temperature was 30 °C, and the testing mode was CR at a shear rate of 100/s. Each viscosity test experiment was sustained for 300 s until the viscosity values tended to stabilize. A total of 80 viscosity measurements were acquired over the duration of analysis, while the final viscosity was defined as the average values of the last 40 data points. The results of pH and viscosity are shown in Table 1. All the synthesized SC adhesives were sealed and stored at room temperature for at least three days before further research was conducted involving them.

### 2.3. Bond Performance

#### 2.3.1. Manufacture of Plywood

The synthesized SC adhesives were utilized to manufacture three-layer plywood (300 mm × 300 mm), the bond performance of which was evaluated. The moisture content and thickness of the veneers were 9.8–11% and 1.5 mm, respectively. SC adhesives were applied to the core veneer at a spread rate of 140 g/m^2^ for a single veneer surface. The coated veneer was stacked between two uncoated veneers so that the grain direction of both adjacent veneers was perpendicular to each other. All assembled three-layered plywood samples with each SC adhesive were hot-pressed at 190 °C for 7 min.

#### 2.3.2. Shear Strength Measurement

The prepared plywood samples were cut into standard tensile shear test specimens according to China National Standards (GB/T 9846.7-2004). Six plywood specimens (10 cm × 2.5 cm) were cut from each manufactured plywood and were submerged in water at 63 ± 2 °C for 3 h. Then, the tensile shear strengths of the plywood samples were measured at wet conditions at a loading rate of 1.0 mm/min. Each plywood was tested in six replications, and the average values, standard deviations, and average wood failure levels were calculated. Statistical significance was considered for *p* values < 0.05.

### 2.4. Analysis of the Synthesis and Curing Mechanisms

#### 2.4.1. C^13^ Nuclear Magnetic Resonance (NMR) Analysis

C^13^ NMR spectra were acquired on a Bruker AVANCE 600 MHz spectrometer equipped with a 5 mm BBO probe using an inverse gated proton decoupling sequence. An amount of 100 mg of freeze-dried SC adhesive was dissolved in 0.5 mL DMSO-*d6*. Then, the solution was transferred to the Shigemi microtube and characterized at 25 °C. The acquisition parameters were 90° pulse width, a relaxation delay of 1.7 s, and an acquisition time of 1.2 s. A total of 10,000 scans were collected.

#### 2.4.2. Attenuated Total Reflection-Fourier Transform Infrared Spectra (ATR-FTIR) Analysis

ATR-FTIR spectra were acquired to assess chemical changes to (i) uncured SC adhesives (after freeze-drying) (ii) insoluble mass of SC adhesive with optimal synthesis conditions (obtained by curing at 190 °C for 7 min, then boiling in distilled water for 4 h, and finally drying at 60 °C for 15 h). Infrared spectra were obtained using an ATR-FTIR spectrophotometer (Nicolet iS10, Thermo, Waltham, MA, USA), and were recorded with an average of 32 scans at a resolution of 4 cm^−1^.

#### 2.4.3. High-Performance Liquid Chromatography (HPLC) Analysis

The chemical composition of the synthesized SC adhesives from Groups 2 and 3 (without freeze-drying) were measured using Agillent 1260 high-performance liquid chromatography (HPLC; Agilent Technologies Inc., Santa Clara, CA, USA). Before the measurement, the adhesive solutions were diluted 300 times. The HPLC system was equipped with an HPX-87H ion exclusion column (300 mm × 7.8 mm), degasser, pump, and refractive index (RI) detector. HPLC-grade milli-Q water was used as eluent at a flow rate of 0.6 mL/min at a column temperature of 55 °C.

## 3. Results and Discussion

### 3.1. Effects of Synthesis Conditions on Viscosity, pH Values, and Crystallization of SC Adhesives

Table 1 shows the basic information of the SC adhesives. All results were measured after storing for three days at room temperature. The viscosity and pH of the synthesized adhesives of Group 1 decreased by adding citric acid, and crystallization could be observed in sucrose (100/0) and citric acid (0/100) solutions. However, the crystalline components were not observed from the adhesives mixed with sucrose and citric acid, indicating that some reaction occurred during the synthesis treatment, and amorphous solutions formed, which prevented the crystallization [24]. With regard to the change of viscosity, due to the hydrolysis of sucrose, an amorphous substance with high viscosity was formed during the heat treatment [25], hence, the viscosity variation of adhesives showed a positive correlation with the sucrose proportion, although some crystallization could be found in the SC (100/0). In both Groups 2 and 3, the pHs of adhesives under all synthesis conditions were almost identical—this was due to the proportion of citric acid being constant. In contrast, the viscosity of Groups 2 and 3 decreased by increasing synthesis temperature and time, which was possible due to the formation of small molecule compounds (such as the monosaccharide and some conversion products [26]) during the heating process.

### 3.2. Effects of Synthesis Conditions on the Bonding Performance

**Figure 1:**
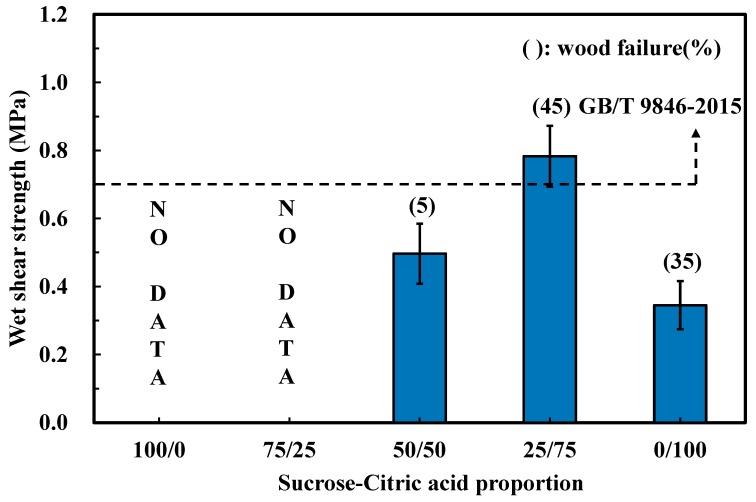
Effects of the mass proportion on the wet shear strength of plywood.

To investigate the effects of different synthesis conditions on the bonding properties, the SC adhesives (prepared by various mass proportions, synthesis temperature, and synthesis times) were utilized to manufacture plywood at 190 °C for 7 min. Figure 1 shows the results of the wet shear strength of the plywood bonded by SC adhesives, which were synthesized with different mass proportions. The plywood manufactured by sucrose only (100/0) and 75/25 adhesives exhibited weak water resistance, and thus, the glue line broke in response to water immersion treatment. With increasing citric acid content to equal and higher than 50% conditions, the plywood showed a certain wet shear strength. The maximum bond strength was achieved by 25/75 mass proportion adhesive (0.78 MPa), which satisfied the China National Standard GB/T 9846-2015. In addition, the value of wood failure of the plywood bonded by 25/75 adhesives showed a clear increase (45%), which signified that the bonding strength between glue line and the wood surface was promoted. Other than the sucrose only condition, the plywood bonded by citric only (0/100) showed 0.35 MPa wet shear strength and 35% wood failure, indicating that citric acid itself contributed to the bond strength and water resistance of plywood. These results demonstrate that the optimal mass proportion between sucrose and citric acid was 25/75.

**Figure 2:**
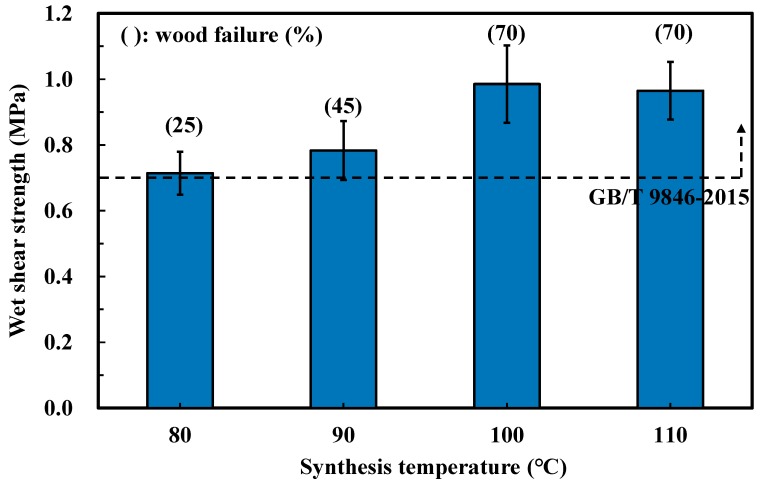
Effects of the synthesis temperature on the wet shear strength of plywood.

Figure 2 shows the results of the wet shear strength of the plywood bonded by the SC (75/25) adhesives, which synthesized at different temperatures. When the synthesis temperature increased from 80 to 100 °C, both wet shear strength and wood failure were promoted, indicating that the bondability of SC adhesives was positively correlated with the synthesis temperature. The maximum value was 0.99 MPa, which was found for the plywood bonded with the adhesive synthesized at 100 °C. However, ANOVA analysis showed no significant difference in the wet shear strength between plywood bonded with SC adhesives synthesized at 100 and 110 °C. This implied that the influence of the synthesis temperature on the bonded strength of SC adhesive levelled off as the temperature exceeded 100 °C. In addition, the wet shear strength of all specimens achieved the requirements of China National Standard GB/T 9846-2015. Judging from the bond performance of each synthesis temperature and considering room for improvement, the synthesis temperature at 100 °C was considered as the optimal condition.

**Figure 3:**
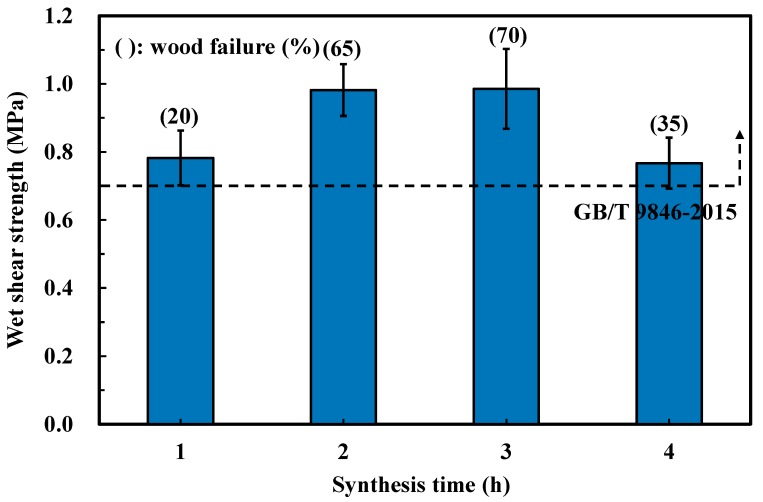
Effects of the synthesis time on the wet shear strength of plywood.

Figure 3 presents the effects of synthesis time on the bond performance of SC adhesives. Comparatively higher wet shear strengths were observed from plywood bonded by adhesives synthesized for 2 h (0.98 MPa) and 3 h (0.99 MPa). The results of the ANOVA analysis indicated that the bond strength between both types of adhesives was almost uniform. However, prolonging the synthesis time to 4 h caused significant decreases of both wet shear strength and wood failure, which was possibly attributed to the chemical transformation during the synthesis and curing processes. Consequently, judging from the bond performance of the plywood bonded with SC adhesives with different synthesis conditions, the optimal mass proportion between sucrose and citric acid, the synthesis temperature, and synthesis time were 25/75, 100 °C, and 2 h, respectively.

### 3.3. Synthesis Mechanism

#### 3.3.1. ^13^C NMR

**Figure 4:**
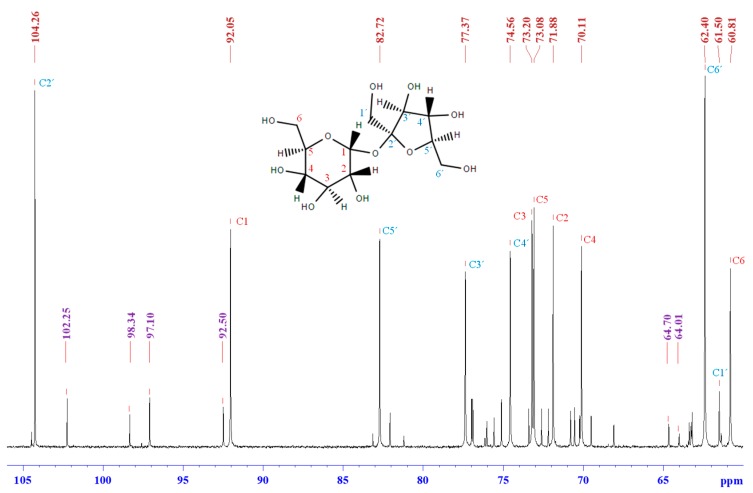
^13^C NMR spectrum of sucrose, prepared by synthesis process, as well as freeze dried and resolved in DMSO-*d_6_*.

To clarify the synthesis mechanism of SC adhesives, the ^13^C NMR spectra of sucrose (100/0), SC adhesive (25/75), and citric acid (0/100), which were prepared at 100 °C for 2 h were determined. The adhesive solutions were freeze-dried prior to the experiments. Figure 4 shows the chemical shift of sucrose (100/0). Two types of peaks can be observed, and the peak with higher intensity corresponded to sucrose [27,28]. In addition, the signals with lesser intensity were considered as the isomerides of glucose and fructose. The characteristic signals of C1 of glucose isomerides were observed at 97.1 ppm (β-d-glucopyranose), 92.5 ppm (α-d-glucopyranose), 102.2 ppm (β-d-glucofuranose), and 98.3 ppm (α-d-glucofuranose) [29,30,31]. With regard to the isomerides of fructose, the peaks at 64.7 and 64.01 ppm were attributed to β-d-fructofuranose and α-d-fructofuranose, respectively [29]. The presence of these signals confirmed the hydrolysis of sucrose during synthesis.

**Figure 5:**
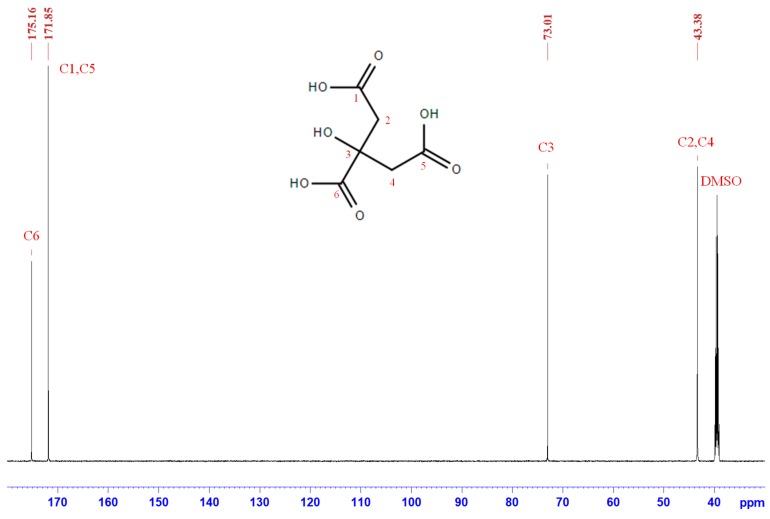
^13^C NMR spectrum of citric acid, prepared by synthesis process, as well as freeze dried and resolved in DMSO-*d_6_*.

**Figure 6:**
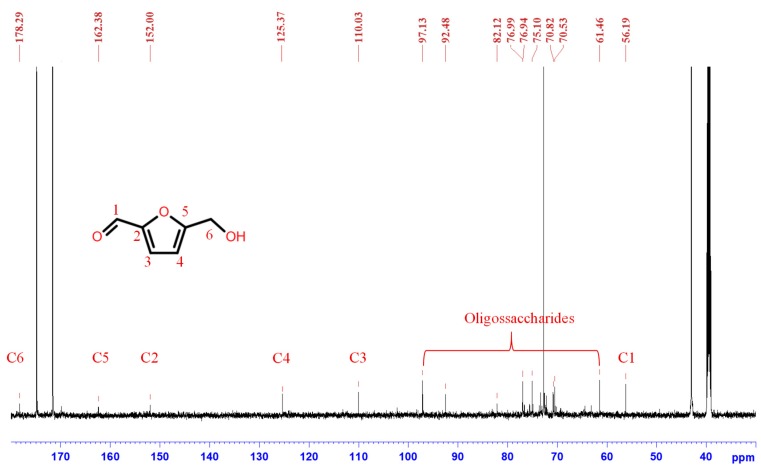
^13^C NMR spectrum of SC adhesive (25/75), prepared by synthesis process, as well as freeze dried and resolved in DMSO-*d_6_.*

The ^13^C NMR spectrum in Figure 5 shows the chemical shifts of citric acid only (0/100), where four high peaks can be attributed to C1–C6 of citric acid [32]. In contrast to sucrose, the specimen containing citric acid only indicated that synthesis treatment did not cause any chemical changes of citric acid. The ^13^C NMR spectra of the 25/75 adhesive, as shown in Figure 6, indicates that the four higher-intensity signals were the result of the existence of citric acid, and the major peak area indicated that the principal compound in the 25/75 synthesized SC adhesive was citric acid. However, compared with the results shown in Figure 4, the characteristic peaks of sucrose disappeared, which implied that sucrose was transformed during the synthesis process. In the lesser intensity signals, the peaks located at 56.19, 152.00, 110.03, 125.37, 162.38, and 178.29 ppm were attributed to C1, C2, C3, C4, C5, and C6 of 5-hydroxymethylfurfural (5-HMF), respectively [33,34,35]. In addition, the resonances concentrated on 60–100 ppm were possibly attributed to oligosaccharides [36,37]. Based on the NMR analysis of the synthesized SC adhesives, the chemical composition of SC adhesive (25/75) differed from that of both sucrose (100/0) and citric acid (0/100). Furthermore, the existence of citric acid and the generation of 5-HMF and oligomers indicated that caramelization of sucrose occurred during the synthesis process, in which, citric acid was considered the catalyst.

#### 3.3.2. HPLC

To investigate the effects of synthesis conditions on the chemical composition of SC adhesives, the contents of glucose and 5-HMF of synthesized SC adhesives of Groups 2 and 3 were measured by HPLC. The results are shown in Table 2. In Group 2, the increase of synthesis temperature led to a reduction of glucose concentration and an increasing of 5-HMF, which was due to the hydrolysis of sucrose and the dehydration of monosaccharide. In Group 3, the content of glucose decreased in response to prolonged synthesis time. However, the concentration of 5-HMF increased from synthesis time at 1–3 h but decreased at 4 h, which was possible because 5-HMF converted to other oligomers, and this was considered as a reason for the reducing of bond strength in Figure 4. Focusing on the change of 5-HMF, clear growth was observed when the synthesis temperature was increased to 100 °C (Group 2) and 2 h (Group 3). Judging from the results of the bonding performance of Groups 2 and 3, the maximum wet shear strength of the plywood bonded with SC adhesives along with the highest concentration of 5-HMF, indicated that the bonding properties of SC adhesives exhibited a positive correlation with the 5-HMF concentration.

### 3.4. Curing Mechanism

**Figure 7:**
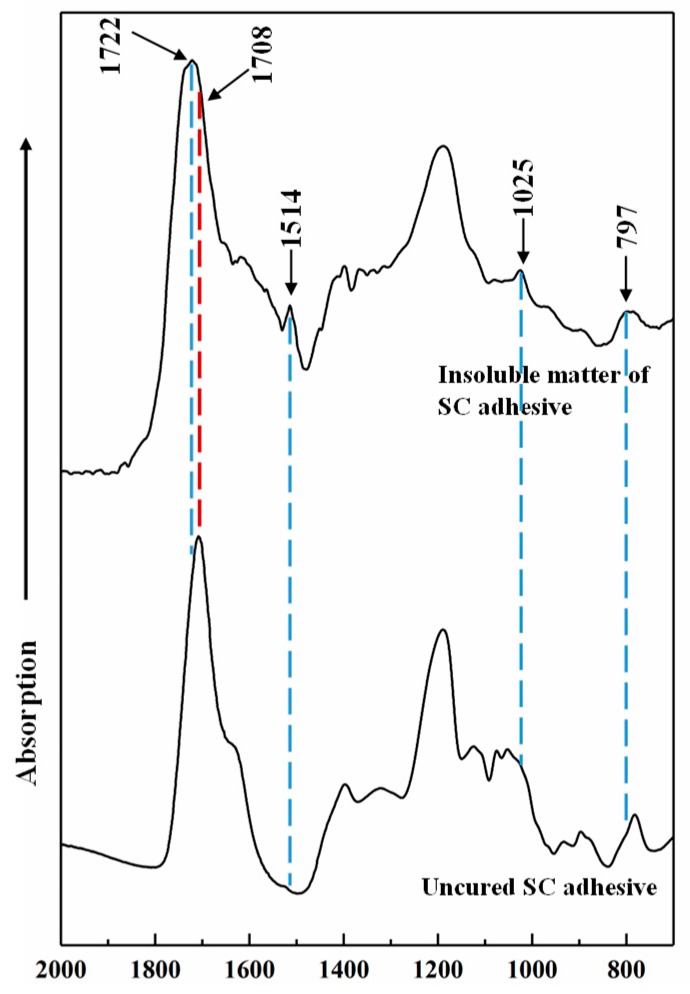
FT-IR spectrum of uncured and insoluble matter of SC adhesive (25/75).

The chemical changes of SC adhesive before and after curing were measured by FT-IR, and the result at the 1800–500 cm^−1^ region is shown in Figure 7. Compared with uncured adhesive, four new peaks were generated and one peak disappeared from the cured adhesive. The new peak was located at 1722 cm^−1^ and could be attributed to C=O stretching derived from carbonyl group and/or ester group [16,38]. The peak that disappeared at 1708 cm^−1^ was due to the C=O of the carboxyl group [39]. Considering the chemical composition of the synthesized SC adhesive and the involved chemical changes, the formation of a novel C=O group was possible due to the reaction between citric acid and 5-HMF. The peaks at 1514 and 797 cm^−1^ were derived from the C=C stretching vibration and the CH=CH of the furan ring [40,41], respectively, indicated that furan compounds participated in the curing reaction. Another new peak located at around 1025 cm^-1^ was identified as the ether linkage C–O–R [42], which possibly formed by the dehydration condensation of furan compounds. In addition, several studies reported that citric acid could react with the hydroxyl group of wood components, which contributed to the bond strength [14,43]. Our results also corroborate this phenomenon (Figure 1). Therefore, the curing mechanism of SC adhesive for the manufacture of plywood should be described via two aspects: one is the dehydration condensation reaction between citric acid and the 5-HMF which derived from the sucrose caramelization, in which, the carbonyl and ether groups formed as cross-linkage; the second is the reaction between citric acid and wood components.

**Figure 8:**
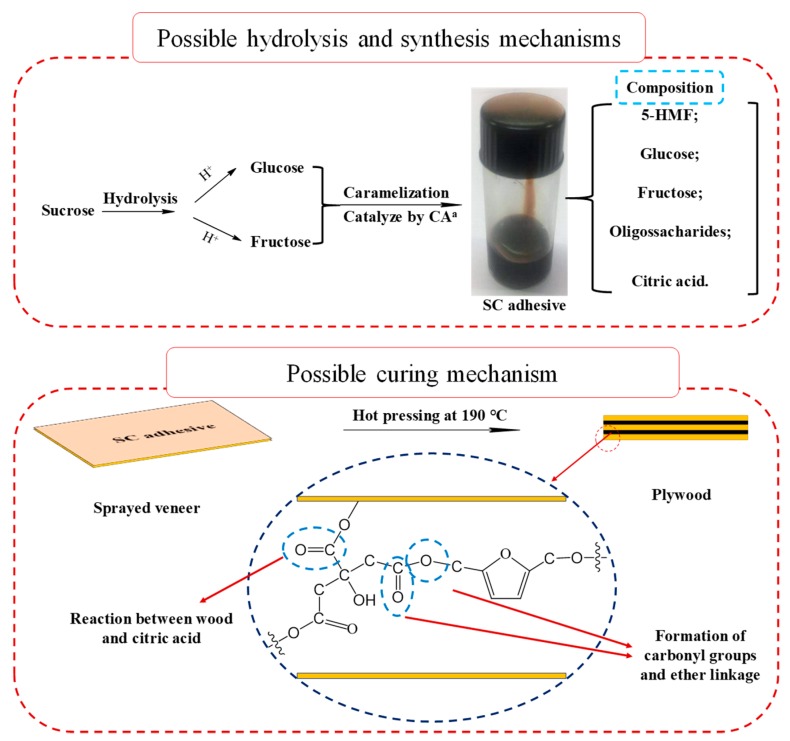
Possible synthesis and curing mechanism of SC adhesive. (^a^ CA: Citric acid).

Based on the chemical analysis above, a possible synthesis mechanism and curing mechanisms are shown in Figure 8. In the synthesis process, 5-HMF and oligosaccharides were formed during the heating treatment, which implied the caramelization reaction as one considered as a synthesis mechanism. During the curing process, the furan ring, carbonyl group, and ether linkage were observed in the cured insoluble matter, and these chemical groups indicated that a dehydration condensation reaction occurred between citric acid and 5-HMF. In addition, the reaction between citric acid and wood constituents also contributed to the bond strength and water resistance of the resultant plywood.

## 4. Conclusions

A novel eco-friendly adhesive was synthesized by combining sucrose and citric acid under heating conditions for incorporation into plywood. The effects of different synthesis conditions (such as mass proportion, synthesis temperature, and synthesis time) on the bonding performance of plywood were investigated. The results of wet shear strength tests indicated that the optimal synthesis conditions of SC adhesive were 25/75 sucrose/citric acid mass proportion, 110 °C, and 2 h. When plywood was bonded with the optimal SC adhesive at 170 °C for 7 min, the wet shear strength achieved the China National Standard GB/T 9846-2015. The results of ^13^C NMR and HPLC showed that 5-HMF and oligosaccharides were generated during the heating treatment. ATR FT-IR indicated the chemical structure change from uncured SC adhesive to cured insoluble matter. Compared with uncured SC adhesive, the furan ring, carbonyl group, and ether linkage were observed in the cured insoluble matter, which indicated that a dehydration condensation reaction occurred between citric acid and 5-HMF. The preliminary research result of this study shows that sucrose and citric acid adhesive could be synthesized and utilized as adhesive for plywood. The curing behaviour, optimal hot-pressing conditions (such as reducing hot-pressing conditions and increase bond performance) will be further investigated in our further research.

## Figures and Tables

**Table 1:** Detailed information of synthesis conditions and results of viscosity, pH values, and precipitation.

Groups	Mass Proportion (Sucrose/CA)	Synthesis Temperature (°C)	Synthesis Time (h)	Design Solid Content (%)	Viscosity (mPa·s)	pH	Whether Contain the Precipitation after 3 Days Storing
	100/0				1770	4.6	Yes
	75/25				1690	1.5	NO
Group 1	50/50	90	3	80	1290	1.2	NO
	25/75				890	1.0	NO
	0/100				20	0.9	Yes
Group 2	25/75	80	3	80	920	1.0	NO
		90			890	1.0	NO
		100			640	0.9	NO
		110			460	0.9	NO
Group 3	25/75	100	1	80	770	1.0	NO
			2		720	1.0	NO
			3		640	0.9	NO
			4		620	0.8	NO

**Table 2:** HPLC results of SC adhesive with 25/75 proportion synthesized under different temperatures and times.

Groups	Sucrose-CA	Synthesis Temperature (°C)	Synthesis Time (h)	Glucose (g/L)	5-HMF (g/L)
Group 2	25/75	80	3	50.7	1.5
		90		46.2	2.9
		100		41.2	8.2
		110		34.5	9.8
Group 3	25/75	100	1	50.4	2.7
			2	47.7	7.8
			3	41.2	8.2
			4	36.4	5.79
